# Diabetes Phenotypes in Patients Presenting a Myocardial Infarction: Progress Towards Precision Medicine?

**DOI:** 10.3390/jpm15090444

**Published:** 2025-09-21

**Authors:** Christelle Lacqua, Arnaud Barbou, Marianne Zeller, Ludwig Serge Aho Glele, Héloïse Adam, Florence Bichat, Jean-Michel Petit, Yves Cottin, Mathieu Boulin

**Affiliations:** 1 Department of Pharmacy, CHU Dijon Bourgogne, 21000 Dijon, France; christelle.lacqua@chu-dijon.fr (C.L.); barbou.arnaud@gmail.com (A.B.); heloise.adam@chu-dijon.fr (H.A.); 2 Cardiology department, CHU Dijon Bourgogne, PEC2 ER 7460, Université Bourgogne Europe, 21000 Dijon, France; marianne.zeller@u-bourgogne.fr (M.Z.); florence.bichat@chu-dijon.fr (F.B.); yves.cottin@chu-dijon.fr (Y.C.); 3 Department of Epidemiology and Infection Control, CHU Dijon Bourgogne, 21000 Dijon, France; ludwig.aho@chu-dijon.fr; 4 Department of Endocrinology, Université Bourgogne Europe, CHU Dijon Bourgogne, INSERM, CTM 1231, PADYS, 21000 Dijon, France; jean-michel.petit@chu-dijon.fr; 5 Department of Pharmacy, Université Bourgogne Europe, CHU Dijon Bourgogne, INSERM, CTM 1231, EPICAD, 21000 Dijon, France

**Keywords:** diabetes, phenotypes, myocardial infarction, precision medicine

## Abstract

**Background/Objectives**: Despite advances in personalized medicine, diabetes classification and management have remained widely unchanged for decades. The aims of the present study were to determine profiles of patients with type 2 diabetes at the time of their myocardial infarction and to assess 1-year cardiovascular events. **Methods**: All type 2 diabetic patients admitted for myocardial infarction in our Coronary Intensive Care Unit between 1 April 2021 and 30 June 2023 were included in this retrospective study. To identify patient profiles, we performed a data-driven cluster analysis based on the *k*-means method according to six characteristics considered as the most relevant in the literature (age at diabetes diagnosis, body mass index, glycated hemoglobin, glutamate decarboxylase antibodies, insulin resistance and beta-cell function). Cox multivariate models were used to identify predictors of 1-year cardiovascular event- and major adverse cardiovascular event-free survivals. **Results:** This study included 250 patients with a median age of 71 years. Our cluster repartition was as follows: 46% patients presented a severe insulin-deficient diabetes, 3% a severe insulin-resistant diabetes, 16% a mild obesity-related diabetes, 33% a mild age-related diabetes, and 2% patients suffered from a severe autoimmune diabetes. In multivariate analyses, the only independent factor for both longer cardiovascular event- and major adverse cardiovascular event-free survival was a higher glomerular function rate (hazard ratio of 0.97 and 0.98 per 1 mL/mn/1.73 m^2^; *p* = 0.01 and *p* = 0.03, respectively). **Conclusions**: This study suggests that the severe insulin-deficient diabetes and mild age-related diabetes pathophysiological phenotypes, easily estimated using insulin resistance and beta-cell function as well as age at diabetes diagnosis, body mass index, and glycated hemoglobin, were more frequent among diabetic patients at the time of their myocardial infarction. In daily clinical practice, caution is needed for patients with a low glomerular function rate, as this was associated with shorter cardiovascular event- and major adverse cardiovascular event-free survival at 1-year.

## 1. Introduction

Diabetes mellitus is a major health problem because of the associated cardiovascular (CV) complications. According to The Emerging Risk Factor Collaboration, the risk of acute CV events (stroke and myocardial infarction (MI)) is two-fold higher in diabetic patients [1].

In an era of personalized medicine, few modifications to diabetes classification and its management have been made for decades. The traditional paradigms of type 2 diabetes only having an onset in adults and type 1 diabetes only in children are no longer accurate, and evidence is accumulating to show that type 1 and type 2 diabetes are heterogeneous diseases in which clinical presentation and disease progression may vary considerably [2]. The paths to β-cell demise and dysfunction are less well defined in type 2 than in type 1 diabetes, but deficient β-cell insulin secretion, frequently in the setting of insulin resistance, appears to be the common denominator. Type 2 diabetes is also associated with insulin secretory defects related to genetic predisposition, epigenetic changes, inflammation, and metabolic stress [2]. Future classification schemes for diabetes will likely focus on the pathophysiology of the underlying β-cell dysfunction, but to date, classifications using the historical terms of type 1 and type 2 diabetes still exist according to the American Diabetes Association and the World Health Organization [2,3]. Among all the authors who have proposed new tools in order to identify higher-risk diabetic patients, the Scandinavian study performed by Ahlqvist et al. is considered as a reference [4]. Their data-driven cluster analysis of six simple variables (glutamate decarboxylase antibodies [GADA], age at diagnosis, body mass index [BMI], glycated hemoglobin [HbA1c], and homeostatic model assessment 2 estimates of β-cell function [HOMA2-B], and insulin resistance [HOMA2-IR]) in 14,755 adult patients with newly diagnosed diabetes identified five replicable clusters of patients with significantly different characteristics and risk of diabetic complications [4]. These included a cluster of very insulin-resistant individuals with higher risk of diabetic kidney disease than the other clusters, a cluster of relatively young insulin-deficient individuals with poor metabolic control (elevated HbA1c), and a large group of older patients with the most benign disease course [4].

A refined diabetes classification could provide a powerful tool to identify at the time of an MI those at higher risk of further CV complications and enable individualized treatment regimens. With this aim, the present study determined the profiles of diabetic patients at the time of their MI and assessed 1-year CV events.

## 2. Materials and Methods

### 2.1. Design

This was a single-center, retrospective study. All consecutive patients admitted for an MI in our French Coronary Intensive Care Unit between 1 April 2021 and 30 June 2023 with a history of type 2 diabetes in their medical file were included in the study. At admission to the Coronary Intensive Care Unit, a standard clinical, biological (including fasting glucose and HbA1c), radiological, and medication review was systematically performed for all patients. In cases of a history of type 2 diabetes mentioned in the medical file before admission, supplemental blood measurements of GADA (Enzyme-Linked ImmunoSorbent Assay) and fasting C-peptide concentrations (electro-chemiluminescence immuno-assay) are also routinely performed. Patients who were newly diagnosed with type 2 diabetes at admission were also included. The sole exclusion criterion was MI with non-obstructive coronary arteries disease (MINOCA) because of its rarity in type 2 diabetic patients. MINOCA is defined as MI with normal or near-normal (less than 50% stenosis) coronary angiography. Its prevalence is only 5–6%, and more than 80% of MINOCA occurs in non-diabetic patients [5]. 

### 2.2. Cluster Analysis

To identify patient profiles, we performed a data-driven cluster analysis based on the *k*-means method. Data entered in the model were inspired by those developed by Ahlqvist et al. [4]: age at diabetes diagnosis, BMI, HbA1c, HOMA2-B and HOMA2-IR based on C-peptide concentrations calculated with the HOMA calculator (University of Oxford, Oxford, UK). To determine the optimal number of clusters, we performed a sensitivity analysis using the Calinski–Harabasz index [6]. Univariate associations between sociodemographic, biological, clinical variables, and clusters were tested by either chi-square or Fisher’s exact test, or non-parametric tests. Among clinical variables, severity of MI was characterized by number and location of lesions and SYNTAX score. The SYNTAX score is basically used to determine the extent and the severity of coronary artery disease and to determine treatment choice based on angiographic data [7]. It was demonstrated that high-sensitivity troponin T was significantly correlated with the angiographic severity of acute coronary syndromes assessed by SYNTAX score [8].

### 2.3. Cardiovascular Events

All CV events were collected over a 1-year period after MI. CV events were divided in Major Adverse Cardiac Events (MACEs) including CV death, MI, stroke, unplanned revascularization, and all other CV events.

CV event-free survival was defined as time from MI to 1st further CV event. MACE-free survival was defined from time to MI to 1st further MACE event. CV event curves (all and MACE) were plotted using the Kaplan–Meier method. For each criterion (all and MACE), a univariate analysis using the log rank test was performed. Variables with a *p*-value < 0.20 were included in the final Cox multivariate models to identify independent predictors. Akaike’s Information Criterion was used to choose the final model. A *p*-value < 0.05 was considered statistically significant. All statistical analyses were performed using the Stata software version 18.0 (Stata corporation, College Station, TX, USA).

## 3. Results

### 3.1. Patients and Clusters

Of the 250 diabetic patients admitted for MI during the study period, 72% were men with a median age of 71 years (range 41–94); 62% presented a non-ST-segment elevation MI (NSTEMI). Further, 48% of patients had a diabetes duration ≥ 10 years and 48% had established atherosclerotic CV disease (ASCVD). The median BMI was 28.6 kg/m^2^ (range 15.8–47.2), and the median HbA1c was 7.1% (range 5.2–12.0).

**Figure 1:**
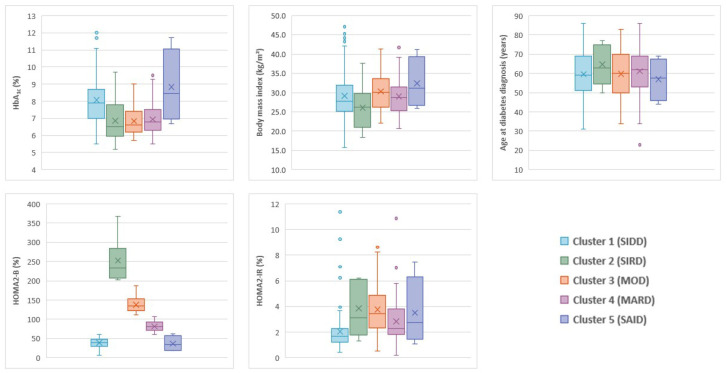
Distributions of HbA1c, body mass index, age at diabetes diagnosis, HOMA2-B, and HOMA2-IR. HbA_1c_ indicates glycated hemoglobin, HOMA2-B indicates homoeostatic model assessment 2 estimates of β-cell function, HOMA2-IR indicates homoeostatic model assessment 2 estimates of insulin resistance, MARD indicates mild age-related diabetes, MOD indicates mild obesity-related diabetes, SAID indicates severe autoimmune diabetes, SIDD indicates severe insulin-deficient diabetes, and SIRD indicates severe insulin-resistant diabetes.

Cluster 1, including 115 (46%) patients, was characterized by either low insulin secretion (low HOMA-2B index) or resistance (low HOMA2-IR index), and was labeled severe insulin-deficient diabetes (SIDD). Conversely, cluster 2, labeled as severe insulin-resistant diabetes (SIRD), including 9 (3%) patients, was characterized by either high insulin secretion or resistance. Cluster 3, including 39 (16%) patients, was characterized by high BMI and labeled mild obesity-related diabetes (MOD). The 83 (33%) patients in cluster 4 were diagnosed with diabetes at an older age than others, without any specific abnormalities. Cluster 4 was labeled mild age-related diabetes (MARD). Cluster 5, including 4 (2%) patients with positive GADA detected at admission, was labeled severe autoimmune diabetes (SAID) (Figure 1).

Patients from cluster 5 were then excluded from analysis because of their specificity (considered as type 1 diabetes/latent autoimmune diabetes) and their very low frequency. There were significant differences in terms of age, gender, tobacco and glomerular function rate (GFR) between the four clusters. Patients from cluster 2 (SIRD) seemed older, with a higher risk of kidney disease (lowest GFR and highest values of urinary albumin/creatinine ratio). Conversely, only very slight differences were observed in terms of blood pressure and low-density lipoprotein cholesterol (LDLc) between the four clusters (Table 1).

Regarding MI characteristics, the STEMI/NSTEMI frequency was significantly different between the four clusters (*p* = 0.04). Patients from cluster 2 (SIRD) only suffered from NSTEMI, whereas patients from other clusters also presented STEMI. There were no significant differences in terms of MI severity between the clusters, but cluster 4 (MARD) and cluster 2 (SIRD) have the highest and lowest SYNTAX score, respectively. Conversely, SIRD and MARD patients mostly presented tritroncular and mono-/bi-troncular lesions, respectively (Table 1).

### 3.2. Cardiovascular Events

At 1 year from MI, at least one CV event had occurred in 46 (19%) patients, divided into 28 patients presenting a MACE and 18 a different CV event. Six patients had presented two or more CV events (two events in four patients, four events in one patient and five events in one patient) over the 1-year period. All CV events are detailed in Table 2.

**Figure 2:**
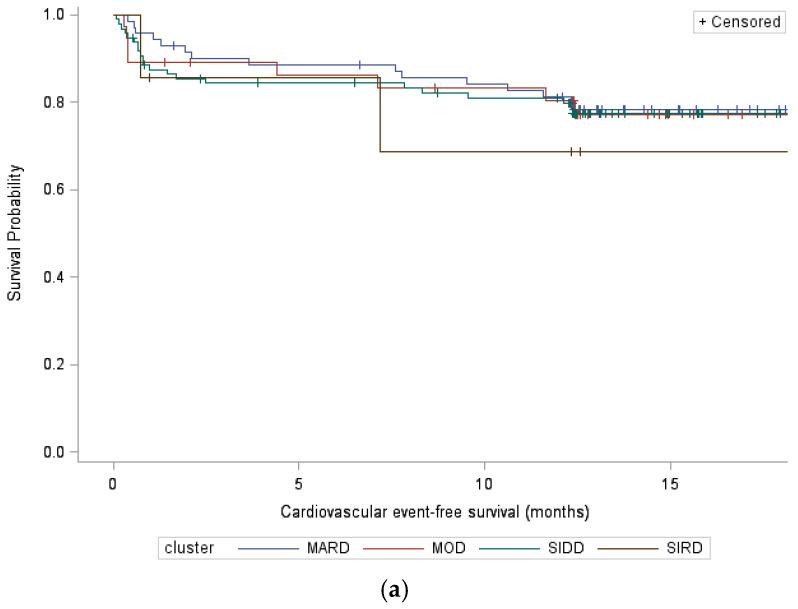

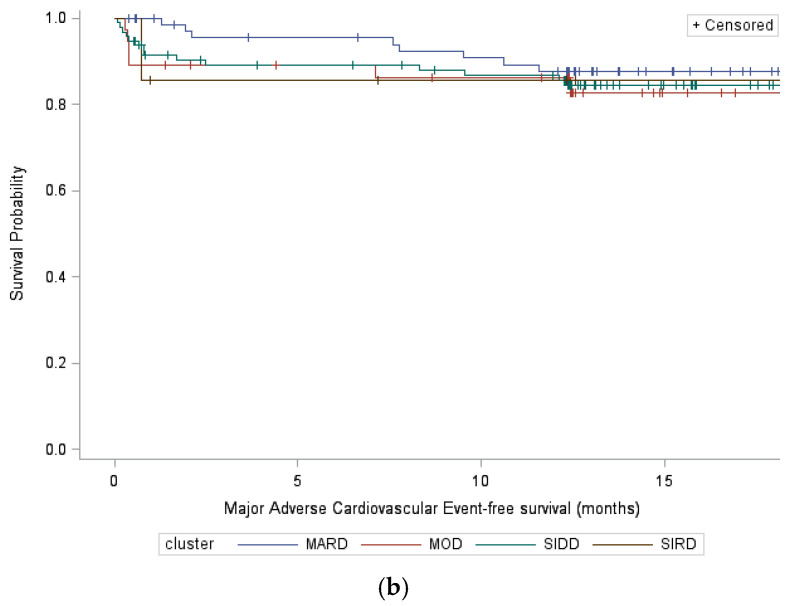
Kaplan–Meier estimated (**a**) cardiovascular event-free survival and (**b**) major adverse cardiovascular event-free survival curves by cluster (log-rank test *p* = 0.92 and 0.86, respectively). MARD indicates mild age-related diabetes, MOD indicates mild obesity-related diabetes, SIDD indicates severe insulin-deficient diabetes, and SIRD indicates severe insulin-resistant diabetes.

There were no significant differences in terms of CV event-free survival between the four clusters (Figure 2a; log-rank test *p* = 0.92). In the univariate analysis, factors that predicted CV event-free survival were ASCVD, GFR, LDL-c, and STEMI. In the multivariate analysis, the only independent factor for longer CV event-free survival was higher GFR (HR 0.98 per 1 mL/mn/1.73 m^2^, according to the chronic kidney disease–epidemiology equation [CKD-EPI]; *p * = 0.01) (Table 3). There were no significant differences in terms of MACE-free survival between the four clusters (Figure 2b; log-rank test *p* = 0.86). In the univariate analysis, factors that predicted MACE-free survival were ASCVD, GFR, and STEMI. In the multivariate analysis, the only independent factor for longer MACE-free survival was higher GFR (HR 0.98 per 1 mL/mn/1.73 m^2^, according to CKD-EPI equation; *p * = 0.03) (Table 3).

There were no significant differences in terms of CV-event free nor MACE-free survivals between clusters. There was a trend towards longer CV-free and MACE-free survivals in SIRD patients and a trend towards longer MACE-free survival in MARD patients.

## 4. Discussion

To our knowledge, this is the first study assessing diabetes phenotypes, MI characteristics, and CV events in a type 2 diabetic population presenting an MI. MI was associated with 79% of the cases and only 2 patient phenotypes, defined as SIDD (46%) and MARD (33%). Our patients’ repartition into clusters is very different from those reported in the literature, in particular by Ahlqvist et al. [2]. The difference could be explained by the fact that we only included patients at the time of their MI, whereas other studies mostly included newly diagnosed diabetic patients, possibly without any ASVCD [4,9,10,11]. This question is very relevant, as diabetic patients with a history of MI are at major risk of newer CV events. Regarding MI characteristics in type 2 diabetic patients, our results are in line with the literature on the results of a majority of NSTEMI, a median age of 71 years, 72% of men, 91% of patients with high blood pressure, and a high prevalence of dyslipidemia and CKD [12,13,14,15], even if our patients had less tritroncular lesions and a lower frequency of low (≤22) SYNTAX score [15].

As reported in the literature, no diabetes clusters were significantly associated with a higher risk of CV events [4,9,16]. At the same time, we observed a trend towards longer CV event-free survival in SIRD patients and longer MACE-free survival in SIRD and MARD patients. Finally, only a low GFR was significantly associated with shorter CV event- and MACE-free survivals. These results are not so easy to explain. First, variables entered in the data-driven cluster model may have separately influenced results. In the case of HbA1c and BMI, the correlation of HbA1c with the occurrence of CV events was less established than for microvascular complications [17], and a higher BMI could exert a positive effect on the long-term clinical outcomes of patients with MI undergoing percutaneous coronary intervention [18]. In 398 diabetic patients presenting NSTEMI, the duration of type 2 diabetes and not HbA1c was significantly associated with a high SYNTAX score and severity of coronary artery disease after controlling for other risk factors [19]. In our study, the mean values for HbA1c and BMI were very close in the different clusters. The mean LDLc value was also very close between the different clusters, whereas it represents a strong predictor for CV events [20,21]. However, it was not significantly associated with shorter CV- nor MACE-event free survival. MARD patients diagnosed with diabetes at an older age than others but without any specific abnormalities did not present shorter MACE-free survival compared to SIDD patients [11,22]. In line with the literature, SIRD patients in our study had the lowest GFR and highest rate of established ASCVD [4,9,10]. Probably due to the low number of them (*n* = 9), SIRD patients did not present shorter CV event- nor MACE-free survival, whereas a lower GFR considered as a major factor of overall CV complications after MI [23] was the only predictor of shorter CV event- and MACE-free survival in our study. In an era of personalized medicine, SIRD patients as well as all patients with CKD should mandatorily and as quickly as possible be treated with a sodium–glucose-like transporter-2 inhibitor, considering the major benefits of this medication class on patient cardiovascular, metabolic, renal outcomes.

Our study has several strengths. In the general population of type 2 diabetic individuals, we present original information on diabetes phenotypes at the time of an MI and their 1-year-associated CV events. To maximize completeness and accuracy over the 1-year period after MI, data were collected from the medical files inside and outside our hospital, with phone calls to patients/families if necessary.

Our study had some limitations. First, it was monocentric and retrospective. However, we included all consecutive patients that presented an MI and had type 2 diabetes over a 27-month period managed at the University Hospital of our regional administrative area of Burgundy (2,805,580 inhabitants according to the 2019 census). Second, except for insulin, we did not evaluate the impact of patient medication (in particular glucose-lowering or lipid-lowering drugs) on cluster repartition or event-free survival analysis. However, the prevalence of statin use before admission was very high and we included dyslipidemia and LDL-c values in our analyses. Finally, even though we performed multivariate analyses to control for confounding as much as possible, we may not have fully adjusted for potential confounding factors such as socio-demographic status, concomitant medications, or comorbidities that could affect both diabetes phenotypes and CV events.

## 5. Conclusions

In conclusion, the SIDD and MARD pathophysiological phenotypes, easily estimated using insulin resistance and beta-cell function as well as age at diabetes diagnosis, BMI, and HbA1c, were more frequent among patients with type 2 diabetes at the time of their MI. We did not identify diabetic phenotypes with higher or lower risks for newer CV events 1 year after an MI. Lower GFR was observed in SIRD patients and was the only independent risk factor for shorter CV event- and MACE-free survival. The retrospective and monocentric characteristics of our study limit these findings. There is an urgent need to conduct large studies to identify personalized profiles based on sociodemographic, clinical, and biological characteristics among patients with type 2 diabetes after an MI and to consequently individualize treatment regimens.

## Figures and Tables

**Table 1:** Patient baseline and myocardial infarction characteristics by cluster (cluster 5 [SAID] excluded).

Cluster	Cluster 1 (SIDD)	Cluster 2 (SIRD)	Cluster 3 (MOD)	Cluster 4 (MARD)	Total	*p*
*n* (%)	115 (47)	9 (3)	39 (16)	83 (34)	246 (100)	
*Baseline characteristics*						
Age (years), median (range)	71 (45–94)	79 (72–90)	70 (48–92)	71 (41–92)	71 (41–94)	**0.03**
Men, *n* (%)	77 (67)	3 (33)	32 (82)	65 (78)	177 (72)	**0.01**
Newly diagnosed diabetes, *n* (%)	8 (7)	0 (0)	2 (5)	10 (12.0)	20 (8)	0.52
High blood pressure, *n* (%)	92 (80)	8 (89)	32 (82)	67 (81)	199 (81)	1.00
Smokers, *n* (%)	34 (30)	0 (0)	18 (46)	38 (46)	90 (37)	**<10^−3^**
Established atherosclerotic cardiovascular disease, *n* (%)	50 (43)	8 (89)	21 (54)	40 (48)	119 (48)	0.05
Prior myocardial infarction, *n* (%)	20 (17)	2 (22)	11 (28)	18 (22)	51 (21)	0.50
Prior stroke, *n* (%)	15 (13)	1 (11)	1 (3)	8 (10)	25 (10)	0.25
Glomerular function rate (CKD-EPI; mL/min/1.73 m^2^), median (range)	84 (6–134)	25 (4–58)	54 (6–128)	74 (13–124)	73 (4–134)	**<10^−3^**
Urinary albumin/creatinine ratio, median (range)	55 (3–3496)	219 (4–5520)	31 (0–2499)	19 (2–6120)	38 (0–6120)	**0.04**
Heart failure, *n* (%)	30 (26)	2 (22)	13 (33)	20 (24)	65 (26)	0.73
Left ventricular ejection fraction (%), median (range)	53 (20–70)	57 (39–70)	54 (23–65)	55 (20–66)	53 (20–70)	0.54
Dyslipidemia, *n* (%)	85 (74)	6 (67)	32 (82)	57 (69)	180 (73)	0.32
Atrial flutter, *n* (%)	13 (11)	2 (22)	8 (21)	14 (17)	37 (15)	0.33
Low-density lipoprotein cholesterol (g/L), median (range)	1.1 (0.4–3.6)	0.9 (0.5–1.6)	1.0 (0.3–1.9)	1.0 (0.4–2.0)	1.0 (0.3–3.6)	0.51
High-density lipoprotein cholesterol (g/L), median (range)	0.4 (0.2–0.8)	0.4 (0.3–0.4)	0.3 (0.2–0.6)	0.4 (0.2–0.7)	0.4 (0.2–0.8)	0.19
Triglycerides (g/L), median (range)	1.5 (0.5–9.5)	2.7 (1.1–3.9)	1.7 (0.6–5.9)	1.4 (0.5–16.1)	1.6 (0.5–16.1)	0.22
Patients with insulin, *n* (%)	37 (32)	3 (33)	7 (18)	15 (18)	62 (25)	0.07
*Myocardial infarction characteristics*						
ST-segment elevation myocardial infarction, *n* (%)	50 (44)	0 (0)	12 (31)	33 (40)	95 (39)	**0.04**
SYNTAX score, median (range) Mild: SYNTAX ≤ 22, *n* (%) Medium: 22 < SYNTAX ≤ 32, *n* (%) Severe: SYNTAX > 32, *n* (%)	13 (0–48) 91 (79) 15 (13) 5 (4)	10 (0–23) 5 (56) 1 (11) 0 (0)	12 (0–61) 31 (79) 1 (3) 3 (8)	14 (0–50) 61 (73) 14 (17) 5 (6)	13 (0–61) 188 (75) 31 (12) 13 (5)	0.75
Lesions, median (range)	2 (0–3)	3 (0–3)	2 (0–3)	2 (0–3)	2 (0–3)	0.63
Lesions, location						0.51
Monotruncular, *n* (%) Bitruncular, *n* (%) Tritruncular, *n* (%)	25 (22) 36 (31) 44 (38)	1 (11) 1 (11) 4 (44)	10 (26) 9 (23) 16 (41)	27 (33) 27 (33) 26 (31)	63 (26) 73 (30) 90 (37)	

CKD-EPI indicates chronic kidney disease epidemiology collaboration, MARD indicates mild age-related diabetes, MOD indicates mild obesity-related diabetes, SIDD indicates severe insulin-deficient diabetes, and SIRD indicates severe insulin-resistant diabetes.

**Table 2:** Cardiovascular events by cluster (cluster 5 [SAID] excluded).

Cluster	Cluster 1 (SIDD)	Cluster 2 (SIRD)	Cluster 3 (MOD)	Cluster 4 (MARD)	Total
*n* (%)	115 (47)	9 (3)	39 (16)	83 (34)	246 (100)
*All CV events, n* (%)	21 (18)	2 (22)	8 (21)	15 (18)	46 (19)
*MACE, n* (%)	14 (12)	1 (11)	6 (15)	7 (8)	28 (11)
Cardiovascular death, *n* (%)	9 (8)	0 (0)	4 (10)	5 (6)	18 (7)
Myocardial infarction, *n* (%)	2 (2)	0 (0)	1 (3)	2 (2)	5 (2)
Ischemic stroke, *n* (%)	3 (3)	1 (11)	0 (0)	0 (0)	4 (2)
Revascularization, *n* (%)	0 (0)	0 (0)	1 (3)	0 (0)	1 (0)
*Other CV events, n* (%)	7 (6)	1 (11)	2 (5)	8 (10)	18 (7)
Unstable angina, *n* (%)	0 (0)	0 (0)	0 (0)	1 (1)	1 (0)
Acute heart failure, *n* (%)	2 (2)	0 (0)	0 (0)	3 (4)	5 (2)
Acute pulmonary edema, *n* (%)	2 (2)	0 (0)	0 (0)	2 (2)	4 (2)
Cardiogenic shock, *n* (%)	1 (1)	0 (0)	0 (0)	0 (0)	1 (0)
Severe hypotension, *n* (%)	1 (1)	0 (0)	0 (0)	0 (0)	1 (0)
Pacemaker implantation, *n* (%)	1 (1)	0 (0)	1 (3)	0 (0)	2 (1)
Chest pain, *n* (%)	0 (0)	1 (1)	1 (3)	0 (0)	2 (1)
Pericarditis, *n* (%)	0 (0)	0 (0)	0 (0)	2 (2)	2 (1)

MACE indicates major adverse cardiovascular events, MARD indicates mild age-related diabetes, MOD indicates mild obesity-related diabetes, SAID indicates severe autoimmune diabetes, SIDD indicates severe insulin-deficient diabetes, and SIRD indicates severe insulin-resistant diabetes.

**Table 3:** Cardiovascular and major adverse cardiovascular events: univariate and multivariate Cox regression models.

	All Cardiovascular Events				Major Adverse Cardiovascular Events			
	Univariate		Multivariate		Univariate		Multivariate	
Variable	HR [CI 95%]	*p*	HR [CI 95%]	*p*	HR [CI 95%]	*p*	HR [CI 95%]	*p*
Cluster								
SIRD vs. SIDD	1.39 [0.34;5.71]	0.65	0.43 [0.08;2.27]	0.32	1.05 [0.13;8.16]	0.96	0.27 [0.03;2.79]	0.27
MOD vs. SIDD	1.04 [0.46;2.36]	0.92	0.62 [0.23;1.66]	0.34	1.17 [0.45;3.06]	0.75	0.66 [0.19;2.24]	0.50
MARD vs. SIDD	0.93 [0.48;1.79]	0.83	0.75 [0.37;1.53]	0.43	0.65 [0.27;1.59]	0.35	0.53 [0.21;1.35]	0.18
Established ASCVD, Yes vs. No	1.60 [0.89;2.88]	**0.12**	1.24 [0.62;2.49]	0.54	1.74 [0.81;3.71]	**0.15**	1.41 [0.65;3.02]	0.38
Glomerular function rate (CKD-EPI; mL/min/1.73 m^2^)	0.99 [0.98;1.00]	**0.003**	0.98 [0.97;1.00]	**0.01**	0.98 [0.97;1.00]	**0.01**	0.98 [0.96;1.00]	**0.03**
Low-density lipoprotein cholesterol (g/L)	0.57 [0.29;1.11]	**0.10**	0.74 [0.33;1.66]	0.47	0.68 [0.29;1.60]	0.38	-	-
NSTEMI vs. STEMI	1.70 [0.90;3.22]	**0.10**	1.51 [0.76;2.97]	0.24	2.00 [0.85;4.70]	**0.11**	1.83 [0.76;4.40]	0.18

ASCVD indicates atherosclerotic cardiovascular disease, CKD-EPI indicates chronic kidney disease epidemiology collaboration, MARD indicates mild age-related diabetes, MOD indicates mild obesity-related diabetes, NSTEMI indicates ST-segment elevation myocardial infarction, SIDD indicates severe insulin-deficient diabetes, SIRD indicates severe insulin-resistant diabetes, and STEMI indicates ST-segment elevation myocardial infarction.

## Data Availability

The data presented in this study are available on request from the corresponding author. The data are not publicly available due to University Hospital of Dijon property rules.

## References

[1] The Emerging Risk Factors Collaboration (2010). Diabetes mellitus, fasting blood glucose concentration, and risk of vascular disease: A collaborative meta-analysis of 102 prospective studies. Lancet.

[2] American Diabetes Association Professional Practice Committee (2025). 2. Diagnosis and Classification of Diabetes: Standards of Care in Diabetes-2025. Diabetes Care.

[3] World Health Organization Diabetes. Key Facts 2024. www.who.int/news-room/fact-sheets/detail/diabetes.

[4] Ahlqvist E., Storm P., Käräjämäki A., Martinell M., Dorkhan M., Carlsson A., Vikman P., Prasad R.B., Aly D.M., Almgren P. (2018). Novel subgroups of adult-onset diabetes and their association with outcomes: A data-driven cluster analysis of six variables. Lancet Diabetes Endocrinol..

[5] Kreimer F., Schlettert C., Abumayyaleh M., Akin I., Hijazi M.M., Hamdani N., Gotzmann M., Mügge A., El-Battrawy I., Aweimer A. (2024). The impact of diabetes mellitus on the outcome of troponin-positive patients with non-obstructive coronary arteries. Int. J. Cardiol. Heart Vasc..

[6] Ekemeyong Awong L.E., Zielinska T. (2023). Comparative Analysis of the Clustering Quality in Self-Organizing Maps for Human Posture Classification. Sensors.

[7] Salimi A., Zolghadrasli A., Jahangiri S., Hatamnejad M.R., Bazrafshan M., Izadpanah P., Dehghani F., Askarinejad A., Salimi M., Bazrafshan Drissi H. (2023). The potential of HEART score to detect the severity of coronary artery disease according to SYNTAX score. Sci. Rep..

[8] Altun B., Turkon H., Tasolar H., Beggı H., Altun M., Temız A., Gazı E., Barutcu A., Bekler A., Colkesen Y. (2014). The relationship between high-sensitive troponin T, neutrophil lymphocyte ratio and SYNTAX Score. Scand. J. Clin. Lab. Invest..

[9] Herder C., Roden M. (2022). A novel diabetes typology: Towards precision diabetology from pathogenesis to treatment. Diabetologia.

[10] Li X., Chen H. (2023). Characteristics of glucolipid metabolism and complications in novel cluster-based diabetes subgroups: A retrospective study. Lipids Health Dis..

[11] Preechasuk L., Khaedon N., Lapinee V., Tangjittipokin W., Srivanichakorn W., Sriwijitkamol A., Plengvidhya N., Likitmaskul S., Thongtang N. (2022). Cluster analysis of Thai patients with newly diagnosed type 2 diabetes mellitus to predict disease progression and treatment outcomes: A prospective cohort study. BMJ Open Diabetes Res. Care.

[12] Humbert O., Noirot E., Leclerc T., Mouhat B., Pommier T., Cochet A., Cottin Y. (2020). Étude et comparaison de la valeur pronostique de différents scores clinique, coronarographique et scintigraphique chez le patient coronarien stable après un syndrome coronarien aigu. Ann. Cardiol. Angiol..

[13] Lee C.D., Folsom A.R., Pankow J.S., Brancati F.L., Atherosclerosis Risk in Communities (ARIC) Study Investigators (2004). Cardiovascular events in diabetic and nondiabetic adults with or without history of myocardial infarction. Circulation.

[14] Gyldenkerne C., Olesen K.K., Thrane P.G., Madsen M., Thim T., Würtz M., O Jensen L., Raungaard B., Poulsen P.L., E Bøtker H. (2020). Diabetes is not a risk factor for myocardial infarction in patients without coronary artery disease: A study from the Western Denmark Heart Registry. Diab Vasc. Dis. Res..

[15] Esper R.B., Farkouh M.E., Ribeiro E.E., Hueb W., Domanski M., Hamza T.H., Siami F.S., Godoy L.C., Mathew V., French J. (2018). SYNTAX Score in Patients With Diabetes Undergoing Coronary Revascularization in the FREEDOM Trial. J. Am. Coll. Cardiol..

[16] Tanabe H., Masuzaki H., Shimabukuro M. (2021). Novel strategies for glycaemic control and preventing diabetic complications applying the clustering-based classification of adult-onset diabetes mellitus: A perspective. Diabetes Res. Clin. Pract..

[17] Gerstein H.C., Miller M.E., Byington R.P., Goff D.C., Bigger J.T., Buse J.B., Cushman W.C., Genuth S., Ismail-Beigi F., Action to Control Cardiovascular Risk in Diabetes Study Group (2008). Effects of intensive glucose lowering in type 2 diabetes. N. Engl. J. Med..

[18] Park S., Kim D.-W., Lee K., Park M.-W., Chang K., Jeong M.H., Ahn Y.K., Chae S.C., Ahn T.H., Rha S.W. (2024). Association between body mass index and three-year outcome of acute myocardial infarction. Sci. Rep..

[19] Biswas S., Mukherjee A., Chakraborty S., Chaturvedi A., Samanta B., Khanra D., Ray S., Sharma R.K., Бисвас С., Мукерджи А. (2022). Impact of plasma glucose and duration of type 2 diabetes mellitus on SYNTAX Score II in patients suffering from non ST-elevation myocardial infarction. Kardiologiia.

[20] Nichols G.A., Joshua-Gotlib S., Parasuraman S. (2013). Independent Contribution of A1C, Systolic Blood Pressure, and LDL Cholesterol Control to Risk of Cardiovascular Disease Hospitalizations in Type 2 Diabetes: An Observational Cohort Study. J. Gen. Intern. Med..

[21] Wan E.Y.F., Fung C.S.C., Yu E.Y.T., Chin W.Y., Fong D.Y.T., Chan A.K.C., Lam C.L.K. (2017). Effect of Multifactorial Treatment Targets and Relative Importance of Hemoglobin A1c, Blood Pressure, and Low-Density Lipoprotein-Cholesterol on Cardiovascular Diseases in Chinese Primary Care Patients With Type 2 Diabetes Mellitus: A Population-Based Retrospective Cohort Study. J. Am. Heart Assoc..

[22] Kahkoska A.R., Geybels M.S., Klein K.R., Kreiner F.F., Marx N., Nauck M.A., Pratley R.E., Wolthers B.O., Buse J.B. (2020). Validation of distinct type 2 diabetes clusters and their association with diabetes complications in the DEVOTE, LEADER and SUSTAIN-6 cardiovascular outcomes trials. Diabetes Obes. Metab..

[23] Weight N., Moledina S., Ullah M., Wijeysundera H.C., Davies S., Chew N.W.S., Lawson C., Khan S.U., Gale C.P., Rashid M. (2024). Impact of Chronic Kidney Disease on the Processes of Care and Long-Term Mortality of Non-ST-Segment-Elevation Myocardial Infarction: A Nationwide Cohort Study and Long-Term Follow-Up. J. Am. Heart Assoc..

